# Infantile epileptic spasm syndrome: predictors of short- and long-term outcomes

**DOI:** 10.3389/fped.2025.1606702

**Published:** 2025-06-12

**Authors:** Mohammed A. Al-Omari, Melissa Chavez-Castillo, Michael R. Miller, Asuri N. Prasad, Maryam Nabavi Nouri

**Affiliations:** ^1^ Department of Pediatrics, College of Medicine, Imam Abdulrahman Bin Faisal University, Dammam, King Fahad Hospital of the University, Al-Khobar, Saudi Arabia; ^2^ Division of Pediatric Neurology, Department of Pediatrics, Hospital Civil Fray Antonio Alcalde, Guadalajara, Mexico; ^3^ Division of Pediatric Neurology, Department of Paediatrics, Schulich School of Medicine & Dentistry, Western University, London, ON, Canada; ^4^ Children's Health Research Institute, London, ON, Canada

**Keywords:** epileptic spasms, outcome predictors, developmental epileptic encephalopathy, infantile epileptic spasm syndrome (IESS), developmental delay

## Abstract

**Introduction:**

Infantile epileptic spasm syndrome (IESS) has significant impact on affected children that affects their future seizure control and neurodevelopmental outcomes. The aim of this study is to identify potential short- and long-term predictors of outcomes in children diagnosed IESS.

**Method:**

This retrospective study evaluated outcomes of seizure control and developmental status in a historical cohort of 60 children with IESS. The predictor variables included: age, treatment regimen, and early treatment response at 14 days, 3 and 6 months on the measured outcomes.

**Results:**

Among the 60 children in the cohort, 75% had identified etiologies: Genetic (40%), Structural (35%), and unknown causes (25%). Treatment interventions included either vigabatrin monotherapy (58.33%) or hormonal therapy with or without vigabatrin (41.67%). Clinical response at 3 and 6 months significantly correlated with good seizure control (*p* = 0.008 and *p* = 0.007, respectively) and favorable developmental outcome (*p* < 0.001) at last follow-up. Logistic regression showed that treatment response at 3 months increased the odds of good seizure control by 7.21 times (95%CI = 1.93–26.91, *p* = 0.003), after adjusting for age, treatment regimen, and etiology. Genetic and structural etiologies were significantly associated with a higher likelihood of developing epileptic encephalopathy (EE), with odds ratios of 11.79 (95% CI = 2.04–68.06, *p* = 0.006) for genetic etiology and 10.21 (95% CI = 1.75–59.65, *p* = 0.010) for structural etiology.

**Discussion:**

Early treatment response at 3 and 6 months strongly predicts favorable seizure and developmental outcomes in IESS, with poor responders at these time points more likely to develop EE. Genetic and structural etiologies significantly influence EE risk, emphasizing the need for early identification, sustained treatment monitoring, and potential targeted interventions for high-risk subgroups.

## 1 Introduction

Infantile epileptic spasms syndrome (IESS) is a diagnostic term adopted by the International League Against Epilepsy (ILAE) to encompass both West syndrome and infants presenting with epileptic spasms (ES) who do not fulfill all the criteria for West syndrome (1). IESS is characterized by presence of ES, the EEG finding of hypsarrhythmia and often by developmental stagnation and/or regression (2). IESS onset typically occurs in children younger than one year, with clinical ES often associated with developmental arrest or regression, though hypsarrhythmia is not always present (3). The estimated incidence of IESS ranges from 2.9 to 4.5 per 10,000 live births (4, 5). While IESS by definition is a form of developmental epileptic encephalopathy (DEE), but outcomes can vary, with some cases resolving and others evolving into different forms of epileptic encephalopathy (EE). Approximately 27% of children with IESS may evolve to Lennox-Gastaut syndrome (LGS), while others may develop different types of epilepsy (6).

Long-term outcomes of IESS, including severity of developmental delay and seizure control, are influenced by promptness of diagnosis and treatment initiation and specific therapeutic approach (1, 7). Up to 24% of surviving patients in one report were found to have normal or only slightly impaired intelligence, as assessed by their educational abilities (8). Several etiologies are known to cause IESS, with structural and genetic etiologies being most common. The underlying etiology has been suggested to be the most important prognostic factor (9). Prior research has shown no significant difference in short-term seizure freedom between two different dosing strategies; i.e., intravenous dexamethasone or methylprednisolone for 3–5 consecutive days, followed by usual-dose (2 mg/kg/day) oral prednisone for 60–90 days with tapering doses for 1–2 months or high-dose (4 mg/kg/day) oral prednisone for 9–11 days with tapering doses for 2–4 weeks, suggesting that the underlying etiology may play a more critical role in treatment response (10). In addition, ACTH is commonly used as a first-line hormonal treatment in many protocols, however, outcome is strongly affected by etiology and the timing of treatment, and no significant differences in terms of efficacy have been documented, though a combination of ACTH and vigabatrin seems to be associated with better long-term outcomes (11). A favorable cognitive outcome has been observed in approximately one quarter of IESS patients and complete seizure freedom in one-third (9). Favorable prognostic factors include early recognition of the ES with prompt treatment, short duration of hypsarrhythmia, prompt treatment of relapses of ES and multifocal epileptic discharges (9). Currently, different treatment protocols for IESS are used, and factors contributing to the successful management of IESS are not well understood. While early intervention improves prognosis, sustained response beyond initial weeks is less explored. The goals of this study were to examine developmental and seizure outcomes post-diagnosis in a historical IESS cohort, aiming to identify predictors—particularly early treatment response at 14 days, 3, and 6 months, to guide management optimization and enhance outcomes and to provide insight into identifying relevant prognostic factors.

## 2 Material and methods

This retrospective, single-center study was conducted at Children's Hospital (CH), London Health Sciences Center, ON, Canada, a tertiary care pediatric epilepsy referral center in Ontario. This study was approved by Institutional Review Board (R# 116581) and a waiver of informed consent was granted. The Pediatric Epilepsy Program database was reviewed between September 1st, 2002 to September 1st, 2020.

Inclusion Criteria:

1. Children, <18 years with a diagnosis of IESS treated under CH pediatric neurology.
2. Availability of clinical data, including seizure control and developmental outcome measures.

Exclusion Criteria:

1. Patients whose treatment was initiated but not completed at CH.
2. Cases with insufficient data precluding reliable outcome analysis.

The primary objective was to identify whether early treatment response (at 14 days, 3 months, and 6 months) would correlate with favorable current seizure control. Secondary objectives included: [1] To identify if early treatment response (at 14 days, 3 months, and 6 months) correlated with favorable current developmental status, and [2] to examine the correlation between early treatment response (at 3 and 6 months) and likelihood of EE persistence later in life.

Seizure control was categorized as either “good” (currently controlled epilepsy, seizure-free for at least one year, or ES-free if diagnosed within the last year) or “poor” (uncontrolled epilepsy, DRE, LGS or persistence of ES). Developmental outcomes were classified as “favorable” (mild or no developmental delay) or “unfavorable” (moderate to severe developmental delays on last clinical evaluation). The severity of developmental delay was determined based on clinical notes, with “mild” defined as functional age more than 66% of chronological age, “moderate” as 34%-66% of chronological age, and “severe” as less than 33% of chronological age as per the consensus amongst authors and extrapolated from other published studies (12, 13). The treatment response was characterized by resolution of ES in response to treatment intervention. The treatment intervention was delineated as vigabatrin therapy alone vs. hormonal therapy with or without vigabatrin. During the study period, the institutional protocol was to initiate treatment with vigabatrin as first-line therapy in all children diagnosed with IESS, regardless of underlying etiology, unless contraindicated or declined by the family. If there was no or inadequate clinical response after two weeks, hormonal therapy was subsequently introduced.

Clinical data for the included children were collected from electronic medical records, encompassing patient details such as age, sex, age at ES onset, treatment initiation, and last follow-up. The data included etiologies were classified into genetic, structural or unknown. Cases with a confirmed genetic diagnosis, such as tuberous sclerosis complex confirmed via genetic testing were categorized under genetic etiology, even when associated with structural abnormalities. Patients presenting with structural abnormalities without a confirmed genetic basis were categorized as structural. This classification acknowledges the evolving nature of genetic testing (e.g., epilepsy panels and whole exome sequencing) and the potential overlap between genetic and structural. Collected data included neuroimaging and genetic test results, EEG findings, and evidence of EE. The study also tracked developmental status and severity of developmental delay at spasm onset and six months post-treatment, the IESS treatment received, clinical response at 14 days, 3 months, and 6 months, as well as current seizure control and developmental status at the latest clinic visit.

## 3 Statistical analysis

Statistical analysis was completed using SPSS v28 (IBM Corp., Armonk, NY, USA). Continuous variables were summarized using means and standard deviations (SD), and categorical variables were summarized using frequencies and proportions. Groups were compared using chi-square tests, and predictors significant at the bivariate level were then included in logistic regression models. SPSS v28 (IBM Corp., Armonk, NY, USA) was used for all analyses, and *p*-values less than 0.01 were considered statistically significant.

## 4 Results

A total of 101 children with IESS were identified, and 60 children (26 females and 34 males) met the inclusion criteria. The remaining 41 children were excluded due to missing clinical data, or their treatment was not guided by staff from our center. The mean age was 92.39 months (Range: 8–216 Months). The mean age of spasm onset was 6.41 months (Range: 1–19 Months).

The age at last clinical follow-up had a mean of 62.43 months. Out of 60 children, an underlying genetic etiology was identified in 24 (40%), structural in 21 (35%), and unknown in the remaining 15 children (25%). EE was evident on the last EEG in 30 children (50%), characterized by persistent epileptiform discharges and features consistent with EE i.e., slow background activity with frequent interictal epileptiform abnormalities. Of the 60 included children, seizure control data available for 51 and developmental status for 52, reflecting variations in data completeness. Good seizure and poor seizure control as defined earlier was achieved in 26 children (43.33%), and 25 children (41.67%) respectively. A favorable developmental outcome as described was observed in 22 children (36.67%) and an unfavorable outcome in 30 children (50%). Neuroimaging was abnormal in 38 children (63.33%). Table 1 shows the basic demographic data and patient characteristics of the included children.

**Table 1:** Demographics and patients characteristics.

Sex (*N*, %)	Male	34 (56.67%)
	Female	26 (43.33%)
Age in months, Mean (SD)	92.39 (66.47)	–
Spasm onset (months), Mean (SD)	6.41 (3.81)	–
Age at last visit (months), Mean (SD)	62.43 (53.47)	–
Etiology (*N*, %)	Genetic	24 (40%)
	Structural	21 (35%)
	Unknown	15 (25%)
Treatment Received (*N*, %)	VGB Only	35 (58.33%)
	Hormonal ± VGB	25 (41.67%)
Evidence of EE on Last EEG (*N*, %)	Yes	30 (50%%)
Good seizure control	Seizure free > 1 year, *N* (%)	19 (31.67%)
	Spasm free < 1 year, *N* (%)	5 (8.33%)
	Controlled generalized epilepsy, *N* (%)	1 (1.67%)
	Controlled Focal epilepsy, *N* (%)	1 (1.67%)
Poor seizure control	ES persist, *N* (%)	7 (11.67%)
	DRE, *N* (%)	9 (15%)
	LGS, *N* (%)	7 (11.67%)
	Uncontrolled Generalized epilepsy, *N* (%)	2 (3.33%)
Developmental status at last assessment (*N*, %)	Normal/Mild Delay	22 (36.67%)
	Moderate/Severe Delay	30 (50%)
Neuroimaging (*N*, %)	Normal	17 (28.33%)
	Abnormal	38 (63.33%)

EE, epileptic encephalopathy; ES, epileptic spasms; DRE, drug resistant epilepsy; LGS, Lennox-Gastaut Syndrome.

Children were categorized into two groups based on the treatment received: (1) Vigabatrin only, the starting dose was 50 mg/kg/day, and escalated every 3 days to a maximum dose of 150 mg/kg/day, comprising 35 children (58.33%), and (2) Hormonal therapy: adrenocorticotropic hormone (ACTH), administered as 75 IU/m^2^ twice daily for two weeks, followed by a taper over two weeks, or prednisolone, the starting dose was 6 mg/kg/day divided TID for two weeks, followed by a taper over 3 weeks, with or without adding vigabatrin to their treatment regimen, 25 (41.67%) children.

No statistically significant difference was identified in treatment response, i.e., resolution of ES, between the 2 treatment groups at 14 days (*p* = 0.34), 3 months (*p* = 0.09), 6 months (*p* = 0.09), and seizure control at last follow-up (*p* = 0.33). Bivariate analysis showed that while early response to treatment at 14 days did not significantly correlate with seizure control at the last follow-up (*p* = 0.35), a significant correlation was observed between treatment response at 3 months (*p* = 0.008) and 6 months (*p* = 0.007) and seizure control at the last follow-up, irrespective of the choice of treatment. Similarly, seizure control at 3 and 6 months significantly correlated with last developmental status (*p* = 0.002 and < 0.001, respectively). Table 2 summarizes the seizure control at different time intervals and its correlation with current seizure control and developmental outcomes.

**Table 2:** The correlation between seizure control at different time intervals with last seizure and developmental status.

Seizure control at:		Last seizure control		*p*-value	Developmental outcome		*p*-value
		Good	Poor		Favorable	Unfavorable	
14 Days	Yes	13 (29.55%)	10 (22.73%)	0.356	15 (33.33%)	6 (13.33%)	0.011
	No	9 (20.45%)	12 (27.27%)		8 (17.78%)	16 (35.56%)	
3 Months	Yes	14 (29.17%)	10 (20.83%)	0.008	14 (29.17%)	8 (16.67%)	0.002
	No	5 (10.42%)	19 (39.58%)		5 (10.42%)	21 (43.75%)	
6 Months	Yes	14 (31.82%)	7 (15.91%)	0.007	16 (36.37%)	4 (9.09%)	<0.001
	No	6 (13.64%)	17 (38.64%)		4 (9.09%)	20 (45.45%)	

Children who later developed EE to respond to IESS treatment at 3 months (*p* = 0.009) and 6 months (*p* < 0.003). Specifically, children with EE had a lower rate of good seizure control (10%) compared to those without EE (40%) during their last clinical assessment (*p* < 0.001).

Additionally, children who later developed EE were less likely to carry favorable developmental status (19.04%) as an outcome compared to children without EE (78.26%) at their last follow-up (*p* < 0.001). Tables 3, 4 summarize the association between IESS treatment response at different time points of the study and their last seizure control, developmental status and later diagnosis of EE.

**Table 3:** The association of epileptic encephalopathy on last EEG with early treatment response at 3 months, 6 months, and current/last seizure control.

Treatment response		3 months			6 months			Current/last seizure control		
		Responders	Non-responders	*p*-value	Responders	Non-responders	*p*-value	Responders	Non-responders	*p*-value
EE on last EEG	Yes	5 (10%)	20 (40%)	0.009	5 (10.87%)	18 (39.13%)	0.003	5 (10.20%)	22 (44.90%)	<0.001
	No	14 (28%)	11 (22%)		15 (32.61%)	8 (17.39%)		19 (38.78%)	3 (6.12%)	

EE, epileptic encephalopathy.

**Table 4:** The association of epileptic encephalopathy on last EEG with developmental status.

Developmental status		Developmental status at 6 months, *N*				
		Normal	Mild delay	Moderate delay	Severe delay	*p*-value
Evidence of EE on last EEG	Yes	1	3	5	15	<0.001
	No	6	12	1	4	
		Current/Last developmental status, *N*				
		Normal	Mild delay	Moderate delay	Severe delay	*p*-value
	Yes	0	3	3	22	<0.001
	No	6	12	1	3	

EE, epileptic encephalopathy.

In a logistic regression model that incorporated multiple predictors including age, treatment regimen, etiology, and clinical response at 3 months, it was observed that treatment response at 3 months increased the odds of good (current/or last) seizure control by a factor of 6 (95% CI, 1.41–25.58, *p* = 0.015) (Table 5).

**Table 5:** Association of seizure control with treatment regimen, age of spasm onset, treatment response, and etiology.

	^a^B (coefficient)	Standard error	*P*-value	Odds ratio (OR)	95% C.I. for OR^b^	
					Lower	Upper
Treatment regimen	−.106	.766	0.89	.899		
Age of spasm onset	.098	.095	0.298	1.103	2.041	68.061
Treatment response	1.792	.740	0.015	6.001	1.749	59.651
Etiology			0.199			

^a^ B, Regression coefficient (log odds).

^b^95% C.I. for OR, Confidence Interval for the odds ratio.

When examining etiology as a predictor of EE, it was found that compared to “unknown” etiology, genetic and structural etiologies were associated with an increased likelihood of EE. The odds ratios (OR) were 11.79 (95% CI = 2.04, 68.06; *p* = 0.006) for genetic etiology and 10.21 (95% CI = 1.75, 59.65; *p* = 0.010) for structural etiology (Table 6). When accounting for age and treatment regimen, etiology was a significant predictor of EE (*p* = 0.04).

**Table 6:** The association of etiology on development of epileptic encephalopathy on last follow up.

	^a^B (coefficient)	Standard error	*P*-value	Odds ratio (OR)	95% C.I. for OR^b^	
					Lower	Upper
Etiological Category			.016			
Genetic Etiology	2.467	.895	.006	11.786	2.041	68.061
Structural Etiology	2.324	.900	.010	10.214	1.749	59.651

^a^ B, Regression coefficient (log odds).

^b^95% C.I. for OR, Confidence Interval for the odds ratio.

Reference treatment category: unknown.

## 5 Discussion

This single-center, retrospective study examined potential predictors of short- and long-term outcomes in children diagnosed with IESS. The main findings were that treatment response at 3 and 6 months positively correlated both with good current seizure control and favorable developmental status. Poor treatment response at 3 and 6 months was a predictor of EE later in life.

The correlation between early treatment response (i.e., 3–6 months after IESS onset) and outcome prognosis were consistent with previous literature suggesting that prompt diagnosis and treatment lead to improved outcomes in children with IESS (7, 14) yet extend focus to sustained response beyond the initial 14-day mark, offering practical insights for clinical monitoring. A prediction model developed from a cohort study indicated that persistent ES or tonic spasms beyond 90 days of onset were significant predictors of poor seizure and developmental outcomes (15). Moreover, moderate or severe MRI abnormalities were also associated with worse prognoses, highlighting the importance of early and accurate diagnostic imaging (15). In our cohort, treatment response at 3 months significantly increased the odds of achieving good seizure control at the most recent follow-up, consistent with findings from the National Infantile Spasms Consortium, which reported that early and sustained responses to treatment improved clinical remission and electrographic resolution of hypsarrhythmia (16).

Treatment regimen (vigabatrin vs. hormonal ± vigabatrin) showed no significant outcome difference (*p* > 0.09), contrasting with UKISS trials where steroid-vigabatrin combinations outperformed monotherapies, but align with findings of a recent study that reported good response on vigabatrin alone (7, 17). This discrepancy may reflect our retrospective design, evolving protocols over 18 years, and smaller sample size.

Our study assessed the treatment response of IESS regardless of the leading time to treatment, or choice of treatment; we demonstrate higher emphasis on clinical response at 3 and 6 months highlighting the importance of sustained treatment response beyond 14-day mark. By adjusting for potential confounding factors such as age, etiology and treatment response, we found that treatment response at 3 months significantly increased the odds of achieving good seizure control at the last follow-up at their most recent clinical assessment compared to those who did not respond well to treatment at 3 months.

We found that children with evidence of persistent EE at their last clinical assessment had a significantly poorer treatment response at 3 and 6 months, were less likely to achieve effective seizure control, and had unfavorable developmental outcomes. This highlights the importance of promptly recognizing the underlying etiological diagnosis and identifying children with poor responses to early treatment, regardless of the treatment protocol used, which increases the likelihood of future diagnosis with EE. Early diagnosis of developmental epileptic encephalopathy (DEE) and understanding that the underlying etiology independently leads to developmental impairment, in which case precision therapies need to be holistic and optimized for these high-risk children (18). In the recent PreVENT trial, early identification and treatment of IESS in those with Tuberous Sclerosis Complex, was not associated with change in cognitive outcomes at 2 years (19). While our results indicate the prognostic importance of early response, they also suggest the need for etiological integration in predicting outcomes and guiding treatment. The analysis further revealed that in patients with IESS, genetic and structural etiologies significantly increase the likelihood of developing EE compared to cases with an unknown etiology. However, when factors such as age and treatment regimen were considered, etiology was no longer a statistically significant predictor of EE. This outcome may be best explained by the small sample size, especially since in most cases of IESS, factors such as treatment regimen are uniformly applied across cases, making it less likely that they would obscure the relationship between etiology and the development of EE. Prior studies looking at neonatal seizures highlights that while specific etiologies (genetic, structural, or metabolic) are associated with different outcomes, including the development of epileptic encephalopathy (EE), the influence of treatment regimens and age at diagnosis can modulate these associations (20).

### 5.1 Study limitations

The retrospective nature of this study introduces several limitations. Retrospective studies rely on the review of existing medical records, which were not originally designed for research purposes. This often results in incomplete or missing data. Although complete seizure control outcomes were available for 51 patients and developmental outcomes for 52 patients, we proceeded with the analysis acknowledging the inherent limitations of retrospective data collection. An ideal cohort would include only those with both outcomes available at ≥2 years post-treatment, this constraint was not feasible given the nature of the available data. Formal developmental assessments were not consistently performed for all children, and data collection methods were not standardized. This can lead to selection bias and recall bias, affecting the reliability of the findings. Additionally, the lack of significant differences observed among the proposed treatment regimens regarding their impact on short-term treatment response or long-term seizure control may have been attributed to the small sample size and the utilization of different treatment protocols during the study period. Genetic testing evolved significantly over the study period, potentially affecting etiology classification consistency. The variability in treatment approaches and the small cohort size limit the generalizability of the results and may obscure potential differences between treatment regimens. Further prospective research is warranted to validate these results and explore targeted therapeutic approaches. Such studies should aim to identify specific biomarkers or genetic factors that may predict response to treatment and guide personalized intervention strategies.

## 6 Conclusion

Our study indicated that children who had poor treatment responses at 3 and 6 months were more likely to have persistent evidence of EE at their last clinical assessment and experienced unfavorable developmental outcomes. While it is well known that prompt diagnosis and optimization of management improves and inform long-term prognosis, our results suggest that etiological diagnoses need to be highly integrated when determining diagnosis and prognosis of IESS. Our findings suggest that potential targeted treatment adjustments may be necessary for IESS patients showing poor early response.

Given that current treatment interventions have not significantly improved outcomes in patients with underlying genetic or metabolic etiologies, there is a growing need for careful curation and personalization of treatment protocols. Early incorporation of precision therapies into treatment plans aligns with the evolving landscape of epilepsy management, where targeted therapies based on underlying pathophysiology are becoming increasingly critical for improving outcomes in children with IESS and other forms of EE.

## Data Availability

The original contributions presented in the study are included in the article/Supplementary Material, further inquiries can be directed to the corresponding author.
