# Supplementary material for: Infantile epileptic spasm syndrome: predictors of short- and long-term outcomes
Source: Front Pediatr. 2025 Jun 12;13:1606702. doi: 10.3389/fped.2025.1606702 (PMC12198225; doi:10.3389/fped.2025.1606702)
Supplement: Supplementary file 1 [file Table1.docx]

| Supplementary Table: Abnormal Neuroimaging Findings in our Cohort | |
| --- | --- |
| Patient 1 | Global hypomyelination, atrophy of supratentorial white matter, corpus callosum, and cerebellum |
| Patient 2 | Multiple non-calcified subependymal nodules; tubers |
| Patient 3 | Dandy Walker continuum: hypoplastic, superiorly rotated cerebellar vermis and prominent 4th ventricle |
| Patient 4 | Corpus callosum hypoplasia, non-specific periventricular FLAIR and T2 hyper-intensities of unknown significance |
| Patient 5 | Prominent CSF extra-axial spaces, without other structural abnormality.  Patient developed diffusion restriction in GP, thalami, midbrain and pontine tegmentum secondary to vigabatrin toxicity |
| Patient 6 | Non-specific volume loss |
| Patient 7 | Agyri-pachygyri, spectrum of lissencephaly |
| Patient 8 | Prominent cisterna magna, mild inferior vermis hypoplasia, general loss of parenchymal volume, mild corpus callosum thickening, and delay myelination |
| Patient 9 | Small cerebellum, hypoplasia of the inferior vermis and pons, reduction in volume of the cerebral white matter, hypoplasia of the corpus callosum and a small right lateral frontal subdural hygroma plus excessive extra-axial CSF spaces around the cerebral hemispheres bilaterally. |
| Patient 10 | Cortical/subcortical tubers both supra- and infratentorial, subependymal nodules, including nodules near the foramina of Monro bilaterally |
| Patient 11 | Numerous subependymal nodules, and multiple cortical tubers |
| Patient 12 | Hydrocephalus with transependymal CSF extension. Diffusion restriction involves regions of the basal ganglia bilaterally in tracts caudally through the midbrain and pons |
| Patient 13 | Multiple cerebral cortical tubers |
| Patient 14 | Multiple cerebral cortical and subcortical tubers. The right posterior parietal lobe cortical/subcortical tuber demonstrates faint enhancement post gadolinium administration. Multiple non-enhancing ependymal nodules, few are calcified |
| Patient 15 | Right open lip schizencephaly |
| Patient 16 | Multiple cortical tubers as well as several subependymal nodules |
| Patient 17 | Atrophy of the basal ganglia and deep white matter. Two foci likely represent microhemorrhages or calcifications. Mild ventriculomegaly. Mildly delayed myelination for age |
| Patient 18 | Subtle white matter high T2 signal in left frontal lobe, with slight volume loss likely a result of previous injury |
| Patient 19 | Partial agenesis of corpus callosum. Nodular heterotropia of frontal grey matter bilaterally. Malformed right lentiform nucleus. Several cystic lesions (1 choroid plexus and 1 inter hemispheric, other small around lateral ventricles and 1 adjacent to foramen of Luschka |
| Patient 20 | Diffusion restriction in supratentorial white matter and along corticospinal tract, laminar necrosis involving central sulcus, insular and parasagittal frontal and parietal lobes. Extensive bilateral cerebral infarcts and generalized white matter volume loss with cystic encephalomalacia |
| Patient 21 | Heterotrophic grey matter in right lateral ventricular atrium and horn. Prominent CSF spaces. Non-specific focus of FLAIR high signal in white matter in sup. medial right frontal lobe |
| Patient 22 | Bitemporal cystic lesions |
| Patient 23 | Focal cortical dysplasia in the left frontal lobe |
| Patient 24 | Enlargement of the ventricular system. The cerebral parenchyma is thinly stretched around the enlarged lateral ventricles. The brainstem is also stretched around the third and fourth ventricles. Very thin band of gliotic or encephalomalacic cerebellar parenchymal tissue is present |
| Patient 25 | Increased fullness in left frontal lobe, insult, and temporal lobe with poor definition of cortical medullary junction and subtle increased FLAIR signal. Thin corpus callous. Suggestive of focal cortical dysplasia |
| Patient 26 | Extensive areas of signal abnormality, swelling and restricted diffusion mainly in the right hemisphere but also involved the left temporal lobe |
| Patient 27 | Left frontal and occipital cortical dysplasia |
| Patient 28 | Large left hemispheric stroke |
| Patient 29 | Bilateral symmetric, confluent, supratentorial white matter signal abnormality. Decreased white matter supratentorial, with ex vacuo prominence of the ventricles compatible with history of perinatal brain injury |
| Patient 30 | Bilateral periventricular nodular gray matter heterotropia. Closed lip schizencephaly. Absent corpus callosum. cyst-like lesion in right cerebellopontine angle |
| Patient 31 | Alobar holoprosencephaly |
| Patient 32 | Widespread leukoencephalomalacia |
| Patient 33 | Right hemispheric porencephalic cyst |
| Patient 34 | Lissencephaly and polymicrogyria |
| Patient 35 | Multiple skull fractures, and brain hemorrhages. Multicystic encephalomalacic changes in the parietooccipital cortex |
| Patient 36 | Numerous hemorrhagic foci in the cerebral white matter which could represent severe acute white matter injury from hypoxic ischemic encephalopathy |
| Patient 37 | Enlarged, dysmorphic ventricular system. Intraventricular hemorrhage |
| Patient 38 | Focus of diminished T2 signal within the posterior perisylvian region on the right side |
